# Importance of optimal rewiring guided by 3-dimensional optical frequency domain imaging during double-kissing culotte stenting demonstrated through a novel bench model

**DOI:** 10.1038/s41598-023-40606-7

**Published:** 2023-08-19

**Authors:** Takayuki Okamura, Kiyotaka Iwasaki, Hongze Lu, Xiaodong Zhu, Tatsuhiro Fujimura, Norika Kitaba, Keisuke Murakami, Ryota Nakamura, Haruki Mitsui, Yusuke Tsuboko, Yousuke Miyazaki, Tetsuya Matsuyama

**Affiliations:** 1 Cooperative Major in Advanced Biomedical Sciences, Joint Graduate School of Tokyo Women’s Medical University and Waseda University, Waseda University, 2-2 Wakamatsucho, Shinjuku, Tokyo, Japan; 2 Division of Cardiology, Department of Medicine and Clinical Science, Yamaguchi University Graduate School of Medicine, Yamaguchi Ube, Japan; 3 Department of Modern Mechanical Engineering, School of Creative Science and Engineering, Waseda University, Tokyo, Japan; 4 Waseda Research Institute for Science and Engineering, Waseda University, Tokyo, Japan; 5 Department of Integrative Bioscience and Biomedical Engineering, Graduate School of Advanced Science and Engineering,, Waseda University, Tokyo, Japan; 6 Institute for Medical Regulatory Science, Comprehensive Research Organization, Waseda University, Shinjuku, Tokyo, Japan

**Keywords:** Interventional cardiology, Biomedical engineering

## Abstract

The usefulness of optical frequency domain imaging (OFDI) guidance on two-stenting at left main bifurcation has not been evaluated. Here, we used a novel bench model to investigate whether pre-defined optimal rewiring with OFDI-guidance decreases acute incomplete stent apposition (ISA) at the left main bifurcation segment. A novel bench simulation system was developed to simulate the foreshortening and overlapping of daughter vessels as well as left main bifurcation motion under fluoroscopy. Double-kissing (DK) culotte stenting was performed using the novel bench model under fluoroscopy with or without OFDI-guidance. In the OFDI-guidance group, if the guidewire did not pass through the pre-defined optimal cell according to the 3-dimensional OFDI, additional attempts of rewiring into the jailed side branch were performed. The success rate of optimal jailed side branch rewiring after implantation of the first and second stent under OFDI-guidance was significantly higher than that under only angio-guidance. After completion of the DK-culotte stenting, the incidence and volume of ISA at the bifurcation segment in the OFDI-guidance group was significantly lower than that in the angio-guidance group. Online 3-dimensional OFDI-guided DK-culotte stenting according to a pre-defined optimal rewiring point might be superior to only angio-guided rewiring for reducing ISA at the bifurcation.

## Introduction

Left main coronary artery disease accounts for 4–9% of patients who undergo invasive coronary angiography^1^. Guidelines recommend revascularization to improve survival in patients with significant left main stenosis^2,3^. Coronary percutaneous treatment of unprotected distal left main bifurcation (LMB) lesions is challenging^1,4^. In general, a provisional approach is recommended according to the 16^th^ expert consensus document of the European Bifurcation Club and clinical guidelines^5^. For complex LMB lesions meeting the criteria of the DEFINITION study^6^, a 2-stent approach, especially the double-kissing (DK) crush technique, however, might be superior to provisional stenting^7,8^. Culotte stenting, on the other hand, does not show superiority to the DK crush technique^9^. DK culotte stenting is reported to be a promising technique based on bench tests^10^. DK approach is thought to reduce stent malapposition and to provide adequate dilatation at side branch (SB) ostium (protective effect on SB ostial stent deformation) and complete lesion coverage for bifurcation^11^. To complete DK culotte stenting, twice distal rewiring is required^10,11^. Previous clinical studies reported a success rate of distal rewiring even in provisional stenting of only 55%–67%^12–14^. In clinical settings, it is sometimes difficult to obtain an ideal angiographic projection to identify the origin of the SB, which increases the chances of SB rewiring, not through the recommended distal cell, but through a proximal cell.

Confirmation of the rewiring point by 3-dimensional (3D) reconstruction of optical frequency domain imaging (OFDI) images during provisional stenting reduced the jailing struts at the bifurcation segment in a randomized clinical trial^14^. We hypothesize that the use of 3D-OFDI guidance for DK culotte stenting may contribute to reducing incomplete stent apposition (ISA) at the LMB segment.

We developed a novel beating LMB system that duplicates the clinical setting with a limited optimal projection viewing of the bifurcation as well as the LMB motion due to the heartbeat. Using the model, we investigated the impact of 3D-OFDI guidance on the incidence and volume of ISA at the bifurcation segment after DK culotte stenting.

## Methods

We conducted 2 studies to investigate the usefulness of OFDI guidance for optimization of LMB culotte stenting. In Study 1, DK culotte stenting was performed using the novel beating LMB system under fluoroscopy with or without OFDI-guidance. The success rate of the optimal rewiring and the incidence of ISA in the bifurcation segment were compared between 2 groups (angio-guidance group vs. OFDI-guidance group). The novel LMB system duplicated the foreshortening and overlapping of daughter vessels as well as LMB motion under fluoroscopy. In Study 2, to investigate the influence of vessel foreshortening, overlapping, and motion on the guidewire manipulations, we compared the success rate of the distal rewiring between the novel beating LMB system and the stationary model placed on the operating table.

### Left main bifurcation model

**Figure 1:**
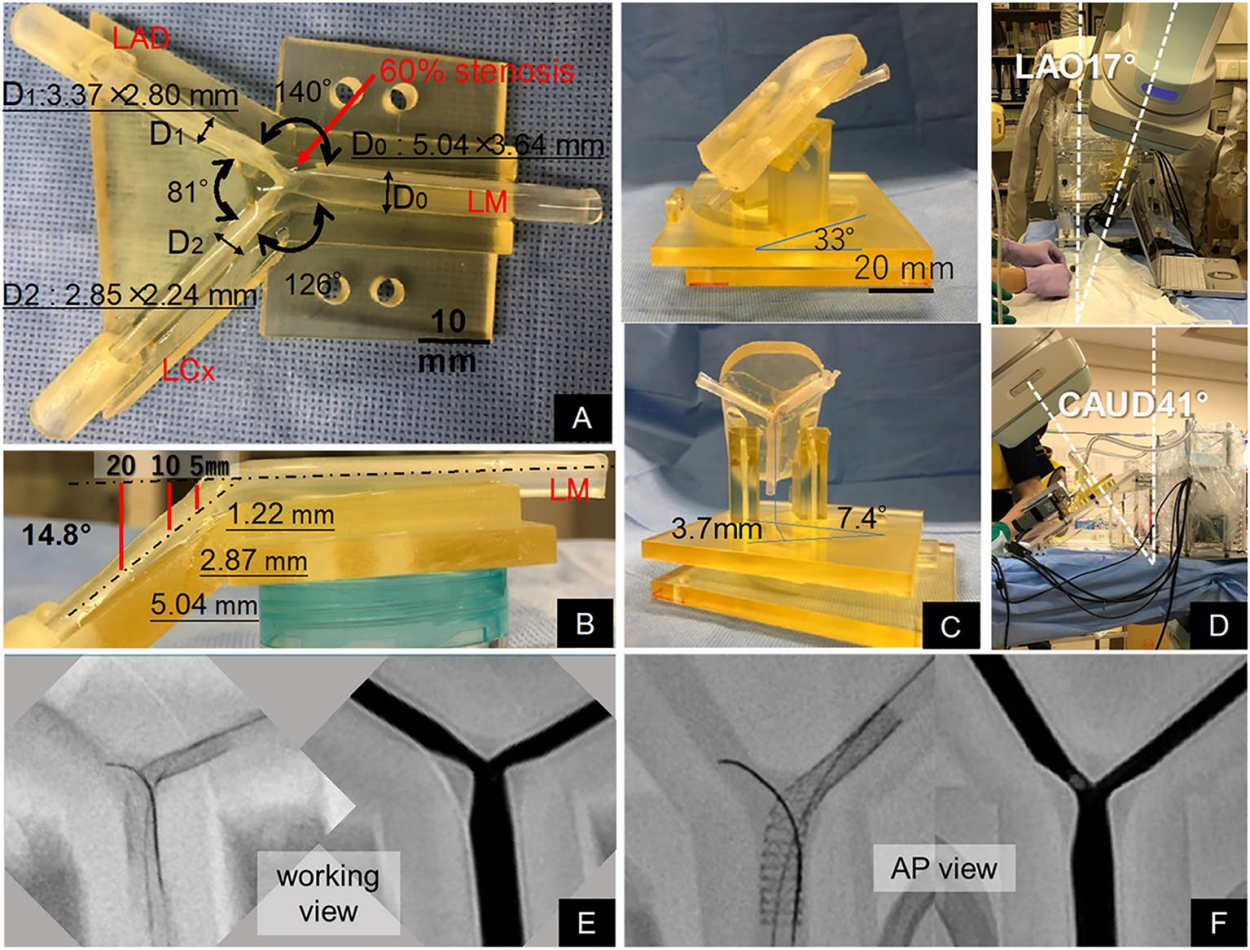
The novel left main bifurcation model. (**A**) Top view, (**B**) side view, (**C**) left main bifurcation model on the base, (**D**) left main bifurcation model with the base on the procedure table and working view projection (left anterior oblique, LAO 17°, caudal 41°), (**E,F**) fluoroscopy and angiography of the left main bifurcation model in working view and anterior–posterior (AP) view.

Morphological data of the LMB from 210 de novo patients (mean age 82.2 ± 7.5 years, 69 men) who underwent coronary computed tomography angiography (CTA) were used to construct the LMB model. From our CT archive from January 2015 to December 2021, we extracted consecutive CTA data that met the following criteria. First, diastolic and systolic CTA data were available. Second, there was no significant stenosis in the LMB segment. Based on each mean value, the 3D LMB model with left main (LM)–left anterior descending (LAD) artery, LM–left circumflex (LCx), and LAD–LCx angles of 140°, 126°, and 81°, respectively, was fabricated. The LMB model had 60% stenosis along the LM, and 50% stenosis at the LAD ostium. The model was constructed with reference vessel diameters of 4.5 mm for LM, 3.2 mm for LAD, and 2.5 mm for LCx. The angle between the LM plane and the daughter vessels was 14.8° (Fig. 1A,B). This study was conducted according to the guidelines of the Declaration of Helsinki and approved by the institutional review board of Yamaguchi University Hospital (H2020-220). The institutional review board of Yamaguchi University has waived the need for informed consent due to the retrospective nature of the study.

### Novel beating left main bifurcation system

A novel beating LMB system simulating the motion of the LMB due to the heartbeat was developed. The LMB model was positioned on the base and driven using the displacement and rotational angle data of the LMB acquired by CTA from 210 patients in systole and diastole. The displacement and rotational angles of the LMB were set to 3.7 mm and 7.4°, respectively (Fig. 1C), under a heart rate of 72 beats/min (See Supplementary Movie S1 online).

### Study 1. Angio-guidance vs. Ofdi-guidance

**Figure 2:**
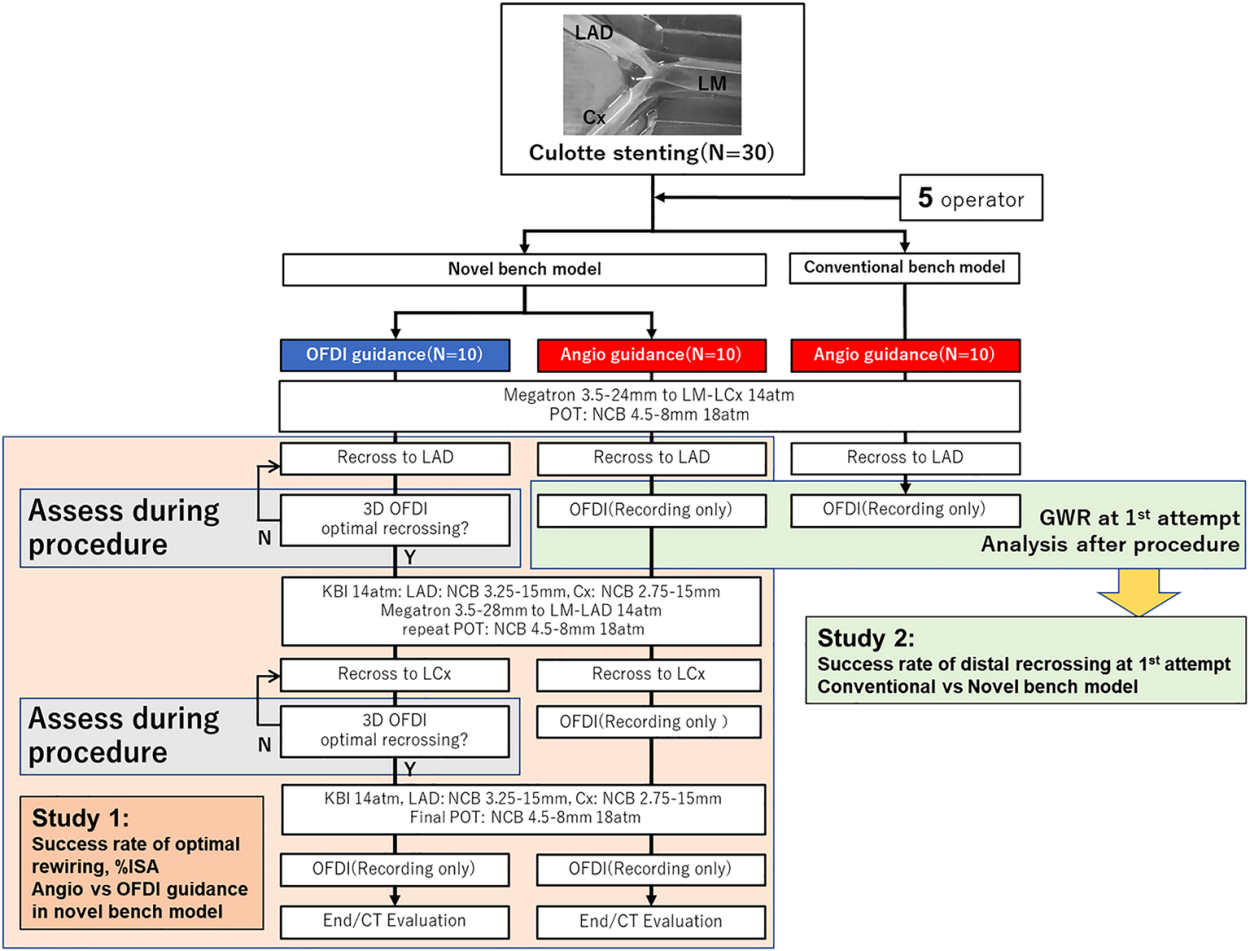
Study design and flow chart. Study 1: Sequence of procedure steps, device used, and dilatation pressure were the same in the angio-guidance and OFDI-guidance groups. 3D-OFDI images were assessed during the procedure by an expert. If the wiring point was not the pre-defined optimal point, the rewiring procedure was repeated. In the angio-guidance group, the procedure steps proceeded under fluoroscopy only. Study 2: Device used, dilatation pressure, and lack of OFDI guidance were the same. Success rate of angiographically distal rewiring after the first attempt in the conventional and novel bench models. LM: left main, LAD: left anterior descending coronary artery, LCx: left circumflex coronary artery, OFDI: optical frequency domain imaging, POT: proximal optimization technique, NCB: non-compliant balloon, 3D: three-dimensional, KBI: kissing balloon inflation, CT: computed tomography, ISA: incomplete stent apposition.

To investigate the usefulness of OFDI guidance during DK culotte stenting for optimal rewiring and stent optimization, procedures were performed on the novel beating LMB system with or without OFDI guidance (n = 10 each, Fig. 2). In the OFDI-guidance group, 3D-OFDI images were reconstructed immediately after pullback. An expert (T.O. and T.F.) judged the appropriateness of the rewiring point according to the pre-defined criteria described below. If the guidewire did not pass through the optimal cell, an additional attempt was made using the information from the OFDI image. In contrast, in the angio-guidance group, the operator was blinded to the OFDI images. Five different operators performed the stenting. All procedures were performed under fluoroscopic working view projection (left anterior oblique, LAO 17° and caudal 41°), which is the best possible viewing projection of this system (Fig. 1D,E). The fluoroscopic time and procedure time were recorded. The success rate of the optimal rewiring defined below for SB dilatation, the incidence and volume of the ISA, minimal stent area and stent expansion ratio as well as the procedure and fluoroscopic time were compared between the study 2 groups.

### DK culotte stent implantation procedures

DK culotte stenting was performed according to the white paper of the European Bifurcation Club and a previously published document^10,11^. The SYNERGY Megatron stent (Boston Scientific Inc., Galway, Ireland) was used in this study^15^. Briefly, after insertion of the guidewire into the LCx, the 3.5-mm stent was deployed from the LM to the LCx, followed by proximal optimization of the LM. A second guidewire was introduced into the LAD, crossing the previously implanted stent at the most distal cell. Manipulation of the guidewire recrossing into the jailed SB was performed using the pullback technique with the appropriate guidewire (ChoicePT, Boston Scientific Inc.) having an appropriately shaped tip curvature^16^. The first kissing balloon inflation (KBI) was then performed. The second 3.5-mm stent was deployed from the LAD to the LM. The stent was then proximally optimized. The guidewire was then introduced into the LCx using the pullback technique. The second KBI was performed. As a final step, proximal optimization was performed.

### OFDI acquisition and rewiring point assessment

**Figure 3:**
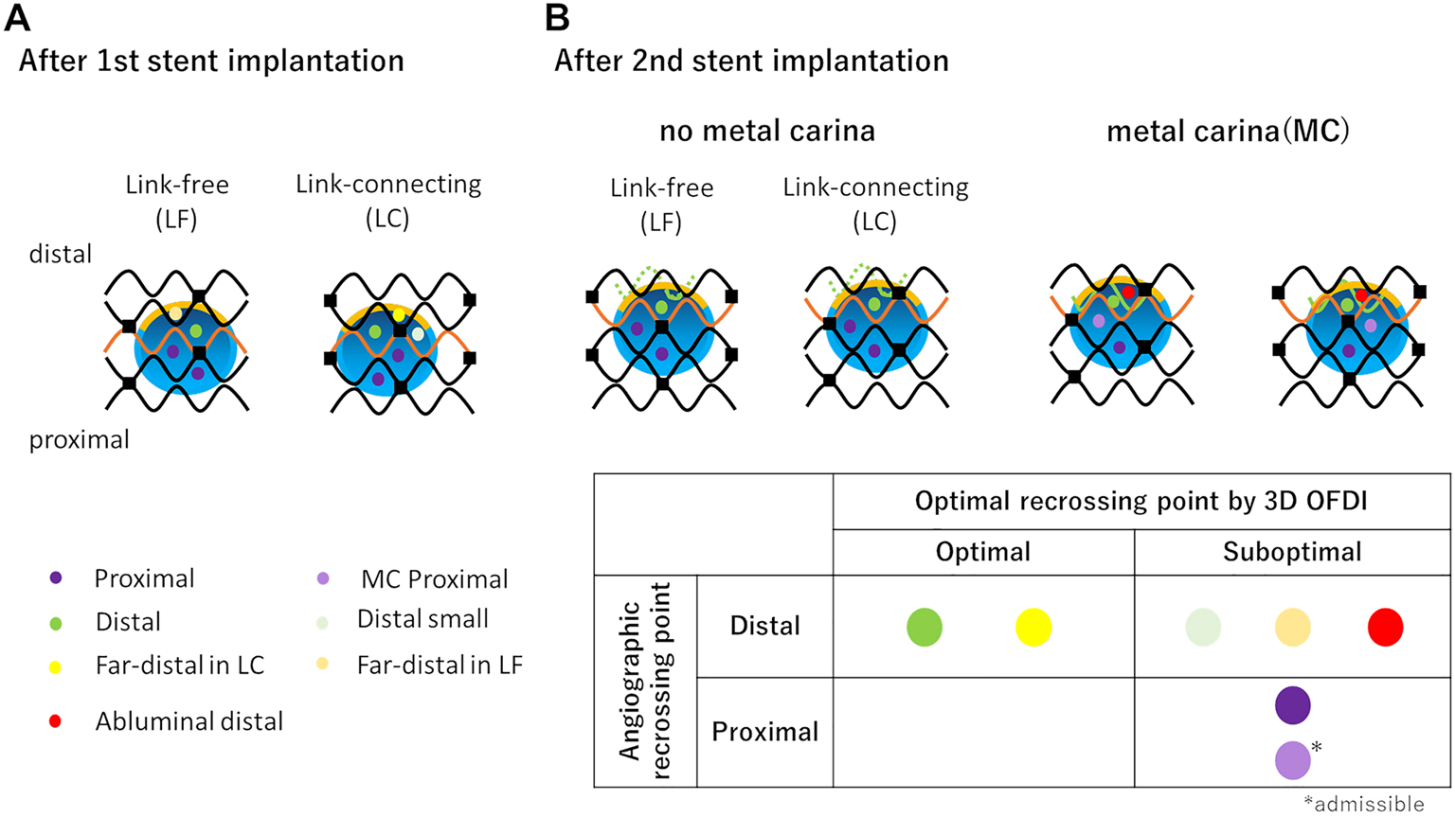
Definition and classification of the optimal rewiring point in the jailed side branch. Blue large circle, yellow arc, and orange wave form indicate the side branch orifice, carina, and crown, respectively, with at least one distal top of the stent hoop located in front of the side branch orifice. Metal carina was defined as a protruding crown, with at least one hoop located in the side branch orifice. Dotted green line indicates protruding crown but no metal carina, while green line indicates a protruding crown. Purple, light purple, green, light green, yellow, light yellow, and red dot indicate rewiring points.

OFDI images were obtained after rewiring to the LAD and LCx, and at the end of the procedure (Fig. 2). The pullback speed was 10 mm/s and the frame rate was 160 frames/s. 3D images were constructed on the OFDI console. Distal rewiring is generally recommended during culotte stenting. According to a previous report^14,17^, the distal cell after the first stent implantation was defined as the cell surrounded by the carina and the crown of the stent. When both larger and smaller distal cells are present, larger cells are optimal (Fig. 3A). A metal carina was defined as the protrusion of at least one strut of the first stent. The optimal location of rewiring after the second stent implantation was defined as shown in Fig. 3B. Angiographically, distal included the far-distal, distal small, and abluminal distal in the present study.

The incidence of ISA at the bifurcation segment was quantified in the post-procedural OFDI pullback. Frame-by-frame cross-sectional images were analysed by counting all individual struts in each frame. ISA, including floating struts at the SB ostium, defined as the separation of at least one stent strut from the vessel wall, was evaluated. Struts were classified as ISA if the distance between the strut marker and lumen contour exceeded the specific strut thickness plus axial resolution of OFDI (14 μm). Strut apposition was assessed in every frame of the bifurcation segment. ISA struts at the bifurcation segment were counted.

Moreover, stent areas were measured at 1-mm intervals in the LM segment [5-mm segment just proximal to the polygon of confluence (POC)] and in the LAD segment (5-mm distal to the carina) on the LAD pullback and in the LCx segment (5-mm distal to the carina) on the LCx pullback. Stent expansion ratios for each segment, where the minimal stent area was divided by the reference lumen area, were compared between the two groups.

### Micro computed tomography assessment

The ISA caused by jailed struts was quantified by micro-CT (TDM 1300-IS, Yamato Scientific Co., Ltd. Japan). The lumen of the bifurcated models was filled with a 70 wt% radiopaque contrast medium (Baritop Sol 150, Sakai Chemical Industry Co., Ltd., Japan) for micro-CT analysis. The 3D structure of the stents was reconstructed using 512 CT slices with a spatial resolution of 0.048 mm.

The ISA volume was defined as the volume between the jailed stent struts and the vessel wall of the bifurcation segment from 2 mm distal to 6 mm proximal to the centre of the polygon of confluence (POC). The bifurcation segment was divided into the following 4 parts: carina, 2 mm distal to the centre of POC; LAD ostium; LCx ostium, 2 mm proximal to the centre of POC; and LM, from 2 to 6 mm proximal to the centre of the POC. The ISA volume was measured in each of the 4 parts.

Stent areas were measured in the LM (5-mm just proximal to the POC), the LAD (5-mm distal to the carina in the LAD), and the LCx (5-mm distal to the carina in the LCx) segments. The minimal stent area was determined as the lowest stent area observed within each segment, as well as at the LAD ostium (from the metal carina to the LAD) and at the LCx ostium (from the metal carina to the LCx).

### Study 2 design

To investigate the impact of the LMB motion on the selection of angiographically distal rewiring, we additionally performed single-crossover stenting (LM-LCx stenting in this study, 10 tests) in an LMB model placed on the operating table without the base. Procedures were performed under optimal fluoroscopic viewing (AP view, Fig. 1F). OFDI images were obtained after the rewiring for documentation of the rewiring points, and the success rates of the distal rewiring after LM-LCx stenting were compared between the 2 models (novel group and conventional group). In addition, quantitative coronary angiography (QCA) was carried out to evaluate vessel foreshortening.

### Statistical analysis

Continuous data are presented as the median and quartile. Comparisons of continuous data with a nonnormal distribution were conducted using the Welch test. The Student-t test was used to compare continuous data with the normal distribution. Categorical variables were compared using the chi-square test. Assumptions for sample size determination were based on past registries. A sample size of 20 tests was determined to provide a power of 80% to show superiority of the OFDI guidance group to the angio-guidance group in Study-1. The assumptions were: (1) incidence of ISA in bifurcation by angio-guidance of 52%^18^, (2) OFDI guidance reduction by ISA by 50%, (3) malapposition rate in bifurcation by OFDI guidance of 24%, based on a previous bench test^10^, 4) a common standard deviation of 15%^14^, and 5) a 5% two-sided level of significance (alpha). The JMP software version 16 (SAS Institute Japan, Co., Ltd., Tokyo, Japan) was used for these analyses. A p value of less than 0.05 was considered statistically significant.

## Results

### Study 1

The success rate of the optimal rewiring and the procedure time are summarized in Table 1. The procedure time and fluoroscopic time were significantly longer in the OFDI-guidance group than in the angio-guidance group.

**Table 1:** Success rate of optimal rewiring and procedure time.

	Angio-guidance N = 10	OFDI-guidance N = 10	p
After the first stent implantation			
Success of optimal rewiring at the first attempt, n(%)	5(50)	7(70)	0.325
Success of optimal rewiring before KBI, n(%)	5(50)	10(100)	
Rewiring point before KBI			
Proximal	4	0	
Distal (Optimal)	5	10	
Distal small	1	0	
Far-distal	0	0	
N of attempts, n	10	14	
After the second stent implantation			
Presence of metal carina	8(80)	5(50)	0.0055
Success of optimal rewiring at the first attempt, n(%)	1(10)	5(50)	
Success of optimal rewiring before KBI, n(%)	1(10)	8(80)	
Rewiring point before KBI			
Proximal	7	2	
Distal (Optimal)	1	8	
Far-distal	0	–	
Abluminal distal	2	–	
No of attempts, n	12	15	
Success of optimal rewiring after both the first and the second stent implantation, n(%)	1(10)	8(80)	0.0055
Procedure time, median (range), minute	25(22–64)	44(36–61)	0.0036
Fluoroscopic time, median (range), minute	10.4(7.5–23.4)	15.1(8.9–23.3)	0.0376

N,n; number, KBI: kissing balloon inflation.

The success rate of optimal rewiring to the LAD at the first attempt after LM-LCx stent implantation was 60% (12/20) overall, and 50% (5/10) and 70% (7/10) for the angio-guidance group and the OFDI-guidance group, respectively. In the OFDI-guidance group, all suboptimal guide wire positions could be changed to the optimal position with no more than 4 additional attempts (Model 3 required 2 additional attempts).

After implantation of the second stent (LM-LAD), OFDI documented metal carina formation in 80% (8/10) of the angio-guidance group and 50% (5/10) of the OFDI-guidance group. The success rate of optimal rewiring at the first attempt was 10% and 50% for the angio- and OFDI-guidance groups, respectively. The number of attempts to recross the guidewire in the LCx was 12 and 15, respectively. In the angio-guidance group, 2 rewiring positions were changed because the device did not pass through the recrossed cell. After 5 additional attempts were made according to the 3D-OFDI image, optimal rewiring was successfully achieved in 80% (8/10) of the OFDI-guidance group. The optimal rewiring point could not be reached in 2 models due to the formation of a metal carina.

**Figure4:**
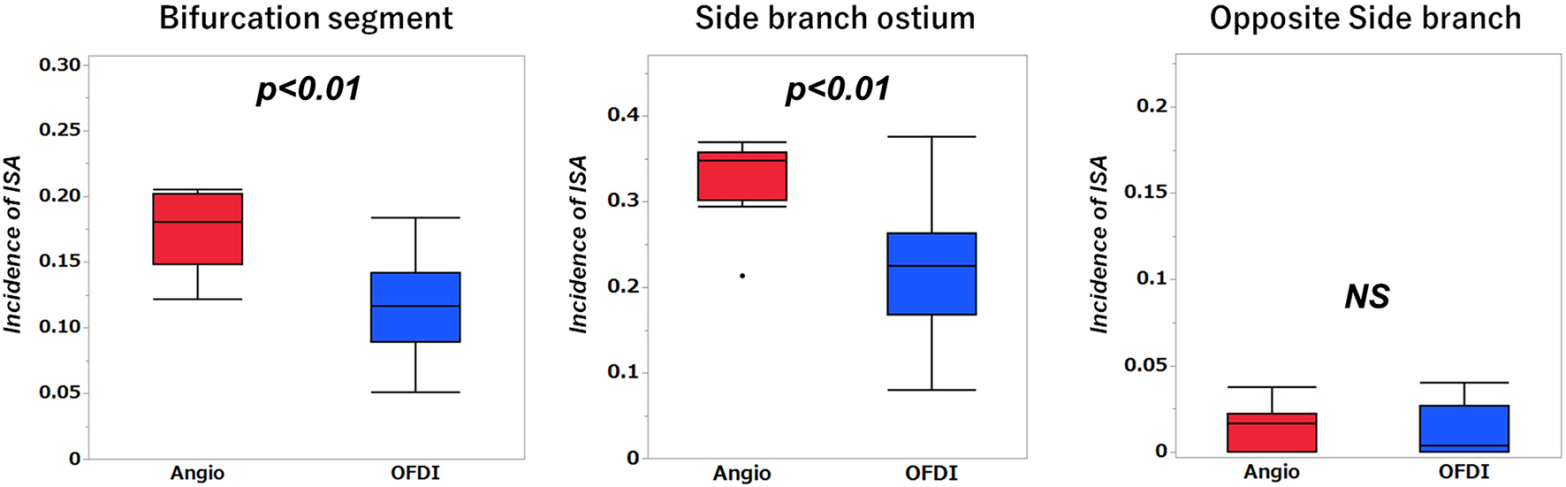
Incidence of incomplete stent apposition by quantitative analysis of optical frequency domain imaging pullback.

After completion of the DK culotte stenting, the incidence of ISA at the bifurcation segment in the OFDI-guidance group was significantly smaller than that in the angio-guidance group (11.6 ± 3.8%, vs. 17.4 ± 2.8%, *p* = 0.0010). Furthermore, OFDI-guidance significantly reduced the incidence of ISA at the LCx ostium compared with angio-guidance (22.2 ± 8.0% vs. 32.8 ± 4.7%, *p* = 0.0020), while the incidences at the opposite side of the SB were small and comparable (1.1 ± 1.5% vs. 1.4 ± 1.2%, *p* = 0.6289, Fig. 4). All 3D-OFDI images obtained during DK culotte stenting are shown in Supplementary Figure S1 and S2 online.

**Figure 5:**
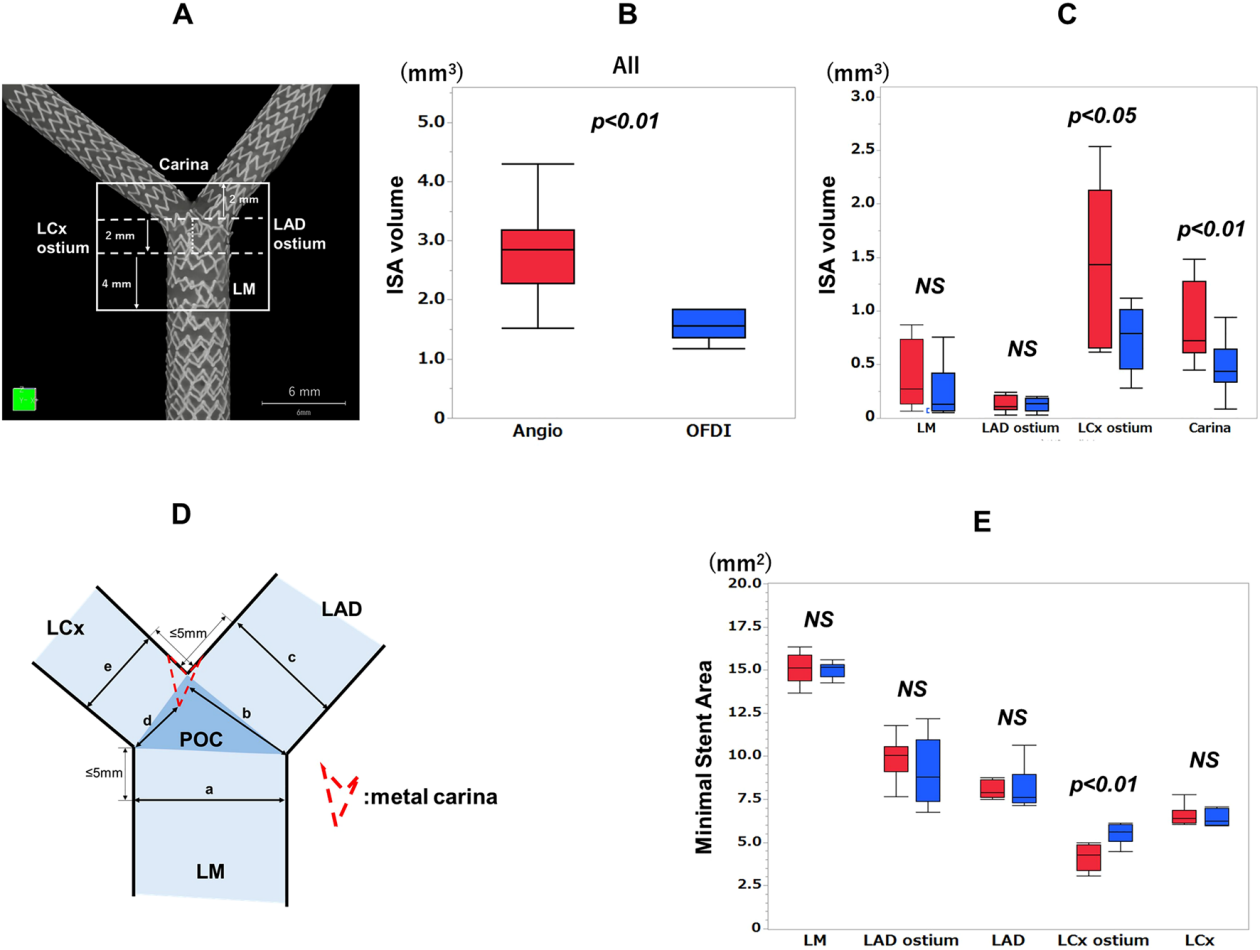
Incomplete stent apposition volume and minimal stent area in bifurcation segment derived by micro computed tomography. Panel (**A**) shows 4 segments for incomplete stent apposition (ISA) volume analysis. (**B**) all segments, (**C**) each segment. LM: left main, LAD: left descending coronary artery, LCx: left circumflex coronary artery, POC: polygon of confluence. Panel (**D**) and (**E**) show minimal stent area analysis by micro-CT. a-e in panel (**D**) are correspond to LM, LAD ostium, LAD, LCx ostium, LCx in panel (**E**), respectively.

The ISA volume detected by the micro-CT analysis is shown in Fig. 5. The ISA volume at the bifurcation segment was significantly lower in the ODFI-guidance group than in the angio-guidance group (1.67 ± 0.49mm^3^ vs. 2.79 ± 0.76mm^3^, *p* = 0.0010, Fig. 5B), particularly at the LCx ostium (0.74 ± 0.30mm^3^ vs. 1.42 ± 0.71mm^3^, *p* = 0.0164) and the carina part (0.47 ± 0.10mm^3^ vs. 0.87 ± 0.10mm^3^, *p* = 0.0099, Fig. 5C).

**Figure 6:**
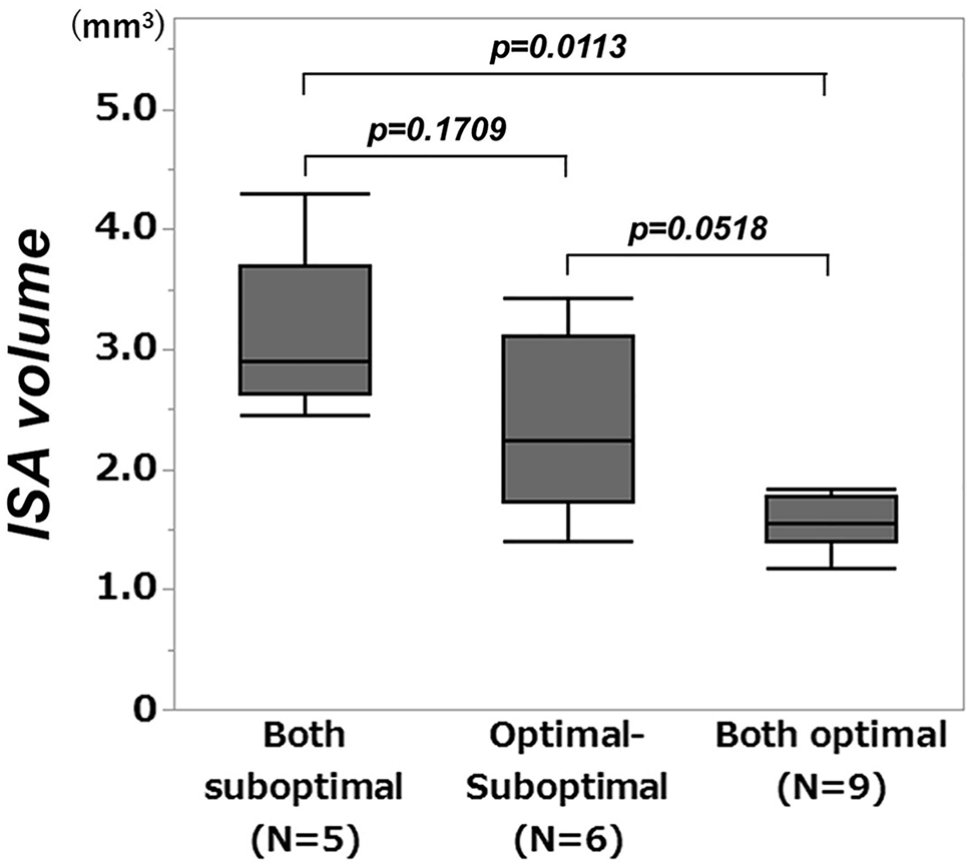
Relation between the sequence of successful optimal rewiring and incomplete stent apposition volume. ISA: incomplete stent apposition, NS: not significant.

The relationship between the sequence of successful optimal rewiring and the ISA volume is shown in Fig. 6. When optimal rewiring of both vessels was achieved, the ISA volume was reduced compared with the others.

Regarding stent expansion after completion of the procedure, OFDI analysis showed no difference in the stent expansion ratios between the two groups. The stent expansion ratio of LCx in the OFDI-guidance group was numerically greater than that in the angio-guidance group (LM: 0.97 ± 0.03 for angio-guidance group vs. 0.98 ± 0.04 for ODFI-guidance group, *p* = 0.3073, LAD: 0.98 ± 0.12 vs. 0.97 ± 0.10, *p* = 0.3847, LCx: 0.86 ± 0.12 vs. 0.98 ± 0.16, *p* = 0.1041, respectively). On the other hand, micro-CT analysis showed the significantly greater MSA at the LCx ostium in the OFDI-guidance group than that in the angio-guidance group (*p* = 0.0012), whereas no difference was observed in the MSA at the other segments between two groups (Fig. 5D,E).

### Study 2

All rewiring positions in the LAD after the first stent implantation were angiographically distal (distal cell 7, distal small cell 2, far-distal cell 1) in the stationary LMB model placed on the operating table, while the success rate of distal rewiring in the novel LMB system was 60% (6/10) (Table 2). Notably, there was no proximal rewiring in the stationary LMB model. As derived by QCA, approximately 10% foreshortening of the LM-LAD distance and a larger distal bifurcation angle were observed in the novel LMB model compared with the actual measurements and the stationary model (see Supplementary Table S1 online).

**Table 2:** Success rate of the distal rewiring.

	Conventional model N = 10	Novel model N = 10	*p*
Rewiring point			
Proximal	0	4(40)	0.0867
Distal	10(100)	6(60)	
Distal large	7(70)	5(50)	
Distal small	2(20)	1(10)	
Far-distal cell	1(10)	0	

## Discussion

The main findings in the present study were as follows: 1) we could succeed in developing the novel beating LMB system which duplicated the clinically relevant foreshortening and overlapping of vessels under a limited projection view and heartbeat motion under fluoroscopy, 2) 3D-OFDI guidance improved the success rate of rewiring into the pre-defined optimal cell and reduced the incidence and volume of ISA at the LMB segment after DK culotte stenting in the beating LMB system, 3) micro CT analysis showed greater MSA at the LCx ostium in the 3D-OFDI guidance than that in the angiography guidance, 4) distal rewiring after the first stent implantation was hindered only with angiography guidance in the novel beating system.

This pre-clinical study evaluated the importance of optimal rewiring under 3D-OFDI guidance during culotte stenting. Several randomized trials have been conducted to assess treatment strategies for LMB. Intracoronary imaging was used for guidance in 40% to 50% of cases, whereas optical coherence tomography (OCT)/OFDI was used in fewer than < 10% of cases^19^. We hypothesized that angiographically distal rewiring is not always optimal in 3D-OFDI, and that OFDI guidance may optimize culotte stenting. To test this and gain new insight, we developed a novel beating LMB system and conducted the stenting.

A recent meta-analysis demonstrated that a 2-stent approach increases cardiac mortality compared with provisional single stenting^20^. The DK crush technique is reported superior to other 2-stent techniques (culotte, crush, T/T-and-protrusion)^9^ and might be effective for complex bifurcation^8^. From a structural point of view, the culotte technique might be considered more “physiologic”, as it ensures full coverage in bifurcation segments (minimal or no neocarina, no triple layer, maintaining tubular stent architecture with fractal deformation only)^10^. Viceconte et al. showed that strut malapposition might be increased in the 2-stent techniques, mainly culotte stenting, compared with single stenting, resulting in increased stent failure^18^. A pathologic study demonstrated that strut malapposition at the bifurcation segment was related to stent failure and optimization by OCT could decrease strut malapposition toward the SB and proximal site^21^. Bench tests by Toth et al. demonstrated that DK culotte stenting was superior to conventional culotte stenting and DK-crush stenting in terms of ISA at the bifurcation^10^. However, its superiority has not been obviously shown in the clinical studies^9^. Uncertainty of guide wire recrossing after second stent deployment is clinically problematic especially in the LAD ostium in the LM bifurcation, which never occurs in DK-crush stenting. Opening the struts of the first stent with a DK process is important to achieve these structural advantages. The DK process might be limited, however, by certain technical restraints in a clinical setting, i.e., stent performance against a patient’s unique bifurcation morphology and failure of distal cell rewiring before KBI. We assumed that the potential risk of failed optimal rewiring before KBI still exists even when using the DK approach and that this could be improved by 3D-OFDI guidance.

Study 2 showed that 30% of rewiring attempts into the LAD after the first stent implantation failed to pass through the angiographically distal cell in the novel beating LMB system even with recommended guidewire manipulation after the proximal optimization technique, whereas all rewiring attempts successfully passed through distally in the stationary LMB model placed on the operating table.

The SB dilatation with non-distal rewiring induces metal carina formation. In clinical settings, the success rate of distal cell rewiring into a jailed SB during provisional stenting is reported to be 55%–67% as evaluated by OCT/OFDI^12–14^. Failed optimal cell rewiring might result from the limited viewing projection of the X-ray angiography system for the SB ostium. Tu et al. reported that an orthogonal view of the LMB could not be observed in approximately 80% of cases due to the mechanical constraints of the X-ray system^22^. In such cases, surrogate viewing projection might be used, but is associated with some degree of vessel foreshortening and overlapping. In the present novel bench system, 11% of foreshortening from the LM to the proximal LAD was observed under the working view projection. Green et al. reported that the mean degree of foreshortening between the working view and the computer-generated optimal view derived by 3D QCA was 10%–15% for the proximal LAD and LCx, similar to our bench model. Another possible factor could be the heartbeat motion at the LMB. Our LMB system cyclically moves with an 0.8-s period, which mimics heartbeat motion. The fluoroscopic rate was 10 frames per second during the procedure — the same condition as in a clinical setting. Heartbeat motion might obscure visualization of the stent structure. Moreover, a patient’s physical build, organs, upper extremities, and breathing motion could interfere with clear visualization of the stent structure under fluoroscopy during guidewire manipulation in a clinical setting. Thus, these differences could hinder the accurate guidewire manipulation, leading to failure of optimal rewiring when performed under fluoroscopy only. We speculate that heartbeat motion might also affect other 2-stenting (i.e. accurate side branch stent deployment in T-stenting, abluminal wiring in crush stenting, and more residual metallic carina in TAP, crush, kissing stenting).

Three-dimensional reconstruction of OCT/OFDI provides detailed information of the bifurcation morphology, jailing configuration by the overhanging strut in front of the SB ostium, and rewiring position^23–25^. The OPTIMUM study demonstrated that selection of the optimal cell by the guidewire before KBI according to the 3D-OFDI could decrease acute ISA at the bifurcation segment compared with angiography guidance only in the provisional stent strategy^14^. Regarding the culotte technique, twice distal rewiring is necessary. The present study revealed that successful optimal rewiring both times significantly decreased the incidence of ISA.

After the second stent implantation, if a metal carina exists, optimal rewiring become quite difficult because the optimal cell is small and there is an increased chance of abluminal rewiring. The incidence of a metal carina was 80% (8/10) in the angio-guidance group, but only 50% (5/10) in the OFDI guidance group. Metal carina formation relates to the risk of abluminal rewiring, leading to un-coverage of the SB ostium. Abluminal rewiring at the first attempt of the second stent implantation was observed in 4 models. The abluminal cell was dilated with a small balloon because it was angiographically distal in the angio-guidance group. In the angio-guidance group, there were 2 models with abluminal rewiring and 7 models required proximal rewiring due to neocarina formation. In contrast, in the OFDI-guidance group, abluminal rewiring was avoided according to the 3D-OFDI information in 2 models. Moreover, in another 2 models, the optimal recrossing point was changed to proximal rewiring or the distal small cell. Consequently, optimal recrossing was achieved in 8 models, resulting in a reduced ISA volume at the bifurcation segment compared with the angio-guidance group. It is often difficult to identify whether or not a recrossing point is optimal only by using angiography. Because the angiographically distal includes far-distal, distal small cell, and abluminal distal, the optimal recrossing point assessed by 3D-OFDI is not always angiographically distal. There is currently no classification for the optimal rewiring position after the second stent implantation, and therefore we classified the optimal rewiring position in the present study. When optimal rewiring was achieved according to the pre-defined classification, the incidence and volume of the ISA was significantly decreased by 3D-OFDI guidance.

Even after optimal rewiring is successfully both times, residual ISA struts and ISA volume remain. The stent design and performance might not be completely suitable for the LMB and may strongly associate with the results of the 2-stent technique. Although optimal rewiring both times under 3D-OFDI guidance decreased the ISA volume, the fluoroscopic time and procedural time were longer in the OFDI-guidance group than in the angio-guidance group. This difference was due to the greater number of attempted guidewire recrossings (2.9 vs. 2.2, *p* = 0.0399). A previous study reported that the total DK culotte stenting procedure duration was 18.3 min^10^, whereas in our study, it was 29.3 min. The increased time was probably caused by the OFDI acquisition (4 times minimally in each case) for documentation and 3 times dilatations for stent deployment and KBI^26^. Prolonged fluoroscopic time results in higher radiation doses delivered to the patients and more contrast volumes used are associated with higher rates of contrast-induced nephropathy^27,28^.

Repeat rewiring and confirmation of the rewiring position by OFDI is complicated and time-consuming. To simplify the procedure and save time, a new dedicated device that can achieve optimal culotte stenting with only angio-guidance may be anticipated.

### Study limitations

We developed a novel beating LMB system that has never before been used. The LMB model represents the average Japanese morphology derived from CTA. Further studies are needed to demonstrate the efficacy of 3D-OFDI guidance in a variety of LM morphologies. In the present study, one type of stent was tested. Stent design and size can influence the results. To minimize the influences of stent performance, we used the same stent sizes and sequences during DK culotte stenting except for 3D-OFDI guidance for optimal rewiring. As a result, confirmation and correction of the recrossing position under OFDI guidance reduced suboptimal rewiring and consequently reduced acute ISA.

Although the clinical impact of reduced ISA at the bifurcation segment by 3D-OFDI guidance cannot be determined in the present study, a previous pathologic report demonstrated that the incidence of ISA struts at the bifurcation and in front of the SB ostium relates to clinical adverse events^21^. Further studies are necessary to investigate whether 3D-OFDI guidance improves clinical outcomes after DK culotte stenting.

## Conclusions

We developed a novel beating LMB system simulating X-ray viewing projection and cardiac motion. Online 3D OFDI-guided DK-culotte stenting according to the pre-defined optimal rewiring point might be superior to angio-guided stenting in terms of acute incomplete stent apposition at the left main bifurcation.

### Supplementary Information

Supplementary Information 1.

Supplementary Video 1.

## Data Availability

The datasets used and/or analysed during the current study available from the corresponding author on reasonable request.

## Abbreviations

LMB: Left main bifurcation
LM: Left main
LAD: Left anterior descending
LCx: Left circumflex
ISA: Incomplete stent apposition
OFDI: Optical frequency domain imaging
SB: Side branch
3D: Three dimensional
KBI: Kissing balloon inflation

## References

[1] Collet C (2018). Left main coronary artery disease: Pathophysiology, diagnosis, and treatment. Nat. Rev. Cardiol..

[2] Lawton JS (2022). 2021 ACC/AHA/SCAI guideline for coronary artery revascularization. J. Am. Coll. Cardiol..

[3] Neumann FJ (2019). 2018 ESC/EACTS guidelines on myocardial revascularization. Eur. Heart J..

[4] Burzotta F (2018). Percutaneous coronary intervention in left main coronary artery disease: the 13th consensus document from the European Bifurcation Club. EuroIntervention.

[5] Albiero R (2022). Treatment of coronary bifurcation lesions, part I: Implanting the first stent in the provisional pathway. The 16th expert consensus document of the European Bifurcation Club. EuroIntervention.

[6] Chen S-L (2014). Impact of the complexity of bifurcation lesions treated with drug-eluting stents. JACC: Cardiov. Intervent..

[7] Chen SL (2017). Double kissing crush versus provisional stenting for left main distal bifurcation lesions: DKCRUSH-V randomized trial. J. Am. Coll. Cardiol..

[8] Zhang J-J (2020). Multicentre, randomized comparison of two-stent and provisional stenting techniques in patients with complex coronary bifurcation lesions: The definition II trial. Eur. Heart J..

[9] Di Gioia G (2020). Clinical outcomes following coronary bifurcation PCI techniques: A systematic review and network meta-snalysis comprising 5,711 patients. JACC Cardiovasc. Interv.

[10] Toth GG (2020). Double-kissing culotte technique for coronary bifurcation stenting. EuroIntervention.

[11] Burzotta F (2020). European Bifurcation Club white paper on stenting techniques for patients with bifurcated coronary artery lesions. Catheter Cardiovasc. Interv.

[12] Alegria-Barrero E, Foin N, Chan PH, Lindsay AC, Di Mario C (2012). Choosing the right cell: guidance with three-dimensional optical coherence tomography of bifurcational stenting. Eur. Heart J. Cardiovasc. Imaging.

[13] Okamura T (2018). Impact of guidewire recrossing point into stent jailed side branch for optimal kissing balloon dilatation: core lab 3D optical coherence tomography analysis. EuroIntervention : J. EuroPCR Collab. Work. Group Interv. Cardiol. Eur. Soc. Cardiol..

[14] Onuma Y (2020). A randomized trial evaluating online 3-dimensional optical frequency domain imaging-guided percutaneous coronary intervention in bifurcation lesions. Circ. Cardiovasc. Interv..

[15] Samant S (2021). Computational and experimental mechanical performance of a new everolimus-eluting stent purpose-built for left main interventions. Sci. Rep..

[16] Burzotta F, De Vita M, Sgueglia G, Todaro D, Trani C (2010). How to solve difficult side branch access?. EuroIntervention.

[17] Okamura T (2014). 3D optical coherence tomography: new insights into the process of optimal rewiring of side branches during bifurcational stenting. EuroIntervention.

[18] Viceconte N (2013). Immediate results of bifurcational stenting assessed with optical coherence tomography. Catheter Cardiovasc. Interv.

[19] Hildick-Smith D (2021). The European bifurcation club left main coronary stent study: A randomized comparison of stepwise provisional vs. systematic dual stenting strategies (EBC MAIN). Eur. Heart J..

[20] Ford TJ (2018). Single- versus 2-stent strategies for coronary bifurcation lesions: A systematic review and meta-analysis of randomized trials with long-term follow-up. J. Am. Heart Assoc..

[21] Mori H (2018). Pathological mechanisms of left main stent failure. Int. J. Cardiol..

[22] Tu S (2012). In vivo assessment of bifurcation optimal viewing angles and bifurcation angles by three-dimensional (3D) quantitative coronary angiography. Int. J. Cardiovasc. Imaging.

[23] Farooq V (2011). New insights into the coronary artery bifurcation hypothesis-generating concepts utilizing 3-dimensional optical frequency domain imaging. JACC Cardiovasc. Interv..

[24] Okamura T (2011). Three-dimensional optical coherence tomography assessment of coronary wire re-crossing position during bifurcation stenting. EuroIntervention.

[25] Onuma Y (2019). Joint consensus on the use of OCT in coronary bifurcation lesions by the European and Japanese bifurcation clubs. EuroIntervention.

[26] Hikichi Y, Umezu M, Node K, Iwasaki K (2017). Reduction in incomplete stent apposition area caused by jailed struts after single stenting at left main bifurcation lesions: Micro-CT analysis using a three-dimensional elastic bifurcated coronary artery model. Cardiovasc. Interv. Ther..

[27] Nikolsky E (2007). An evaluation of fluoroscopy time and correlation with outcomes after percutaneous coronary intervention. J. Invasive. Cardiol..

[28] Tajti P (2021). Association of prolonged fluoroscopy time with procedural success of percutaneous coronary intervention for stable coronary artery disease with and without chronic total occlusion. J. Clin. Med..

