# Supplementary material for: Importance of optimal rewiring guided by 3-dimensional optical frequency domain imaging during double-kissing culotte stenting demonstrated through a novel bench model
Source: Sci Rep. 2023 Aug 19;13:13511. doi: 10.1038/s41598-023-40606-7 (PMC10439901; doi:10.1038/s41598-023-40606-7)
Supplement: Supplementary file 1 — Supplementary Information 1. [file 41598_2023_40606_MOESM1_ESM.docx]

**Supplementary Information**

**Importance of optimal rewiring guided by 3-dimensional optical frequency domain imaging during double-kissing culotte stenting demonstrated through a novel bench model**

Takayuki Okamura, MD, PhD, Kiyotaka Iwasaki, PhD, Hongze Lu, Xiaodong Zhu, PhD, Tatsuhiro Fujimura, MD, PhD, Norika Kitaba, Keisuke Murakami, Ryota Nakamura, Haruki Mitsui, Yusuke Tsuboko, PhD, Yousuke Miyazaki, MD, PhD, Tetsuya Matsuyama, MD.

**Supplementary Movie S1: Novel beating left main bifurcation system**

**Supplementary Figure S1: 3D OFDI image from the angio-guidance group**


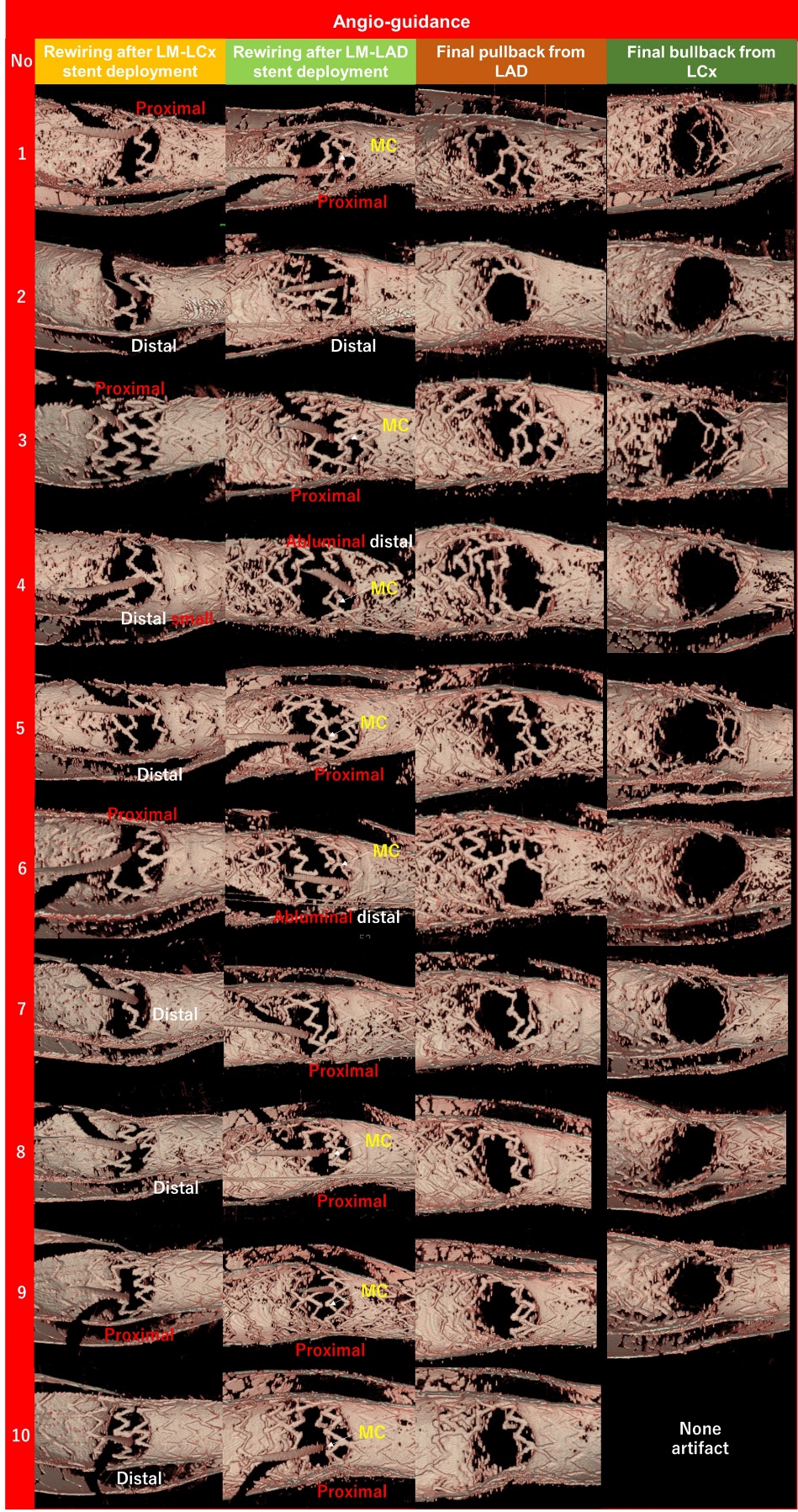


MC: metal carina, other abbreviations are the same as for Figure 2.

**Supplementary Figure S2: 3D OFDI image from the OFDI-guidance group**


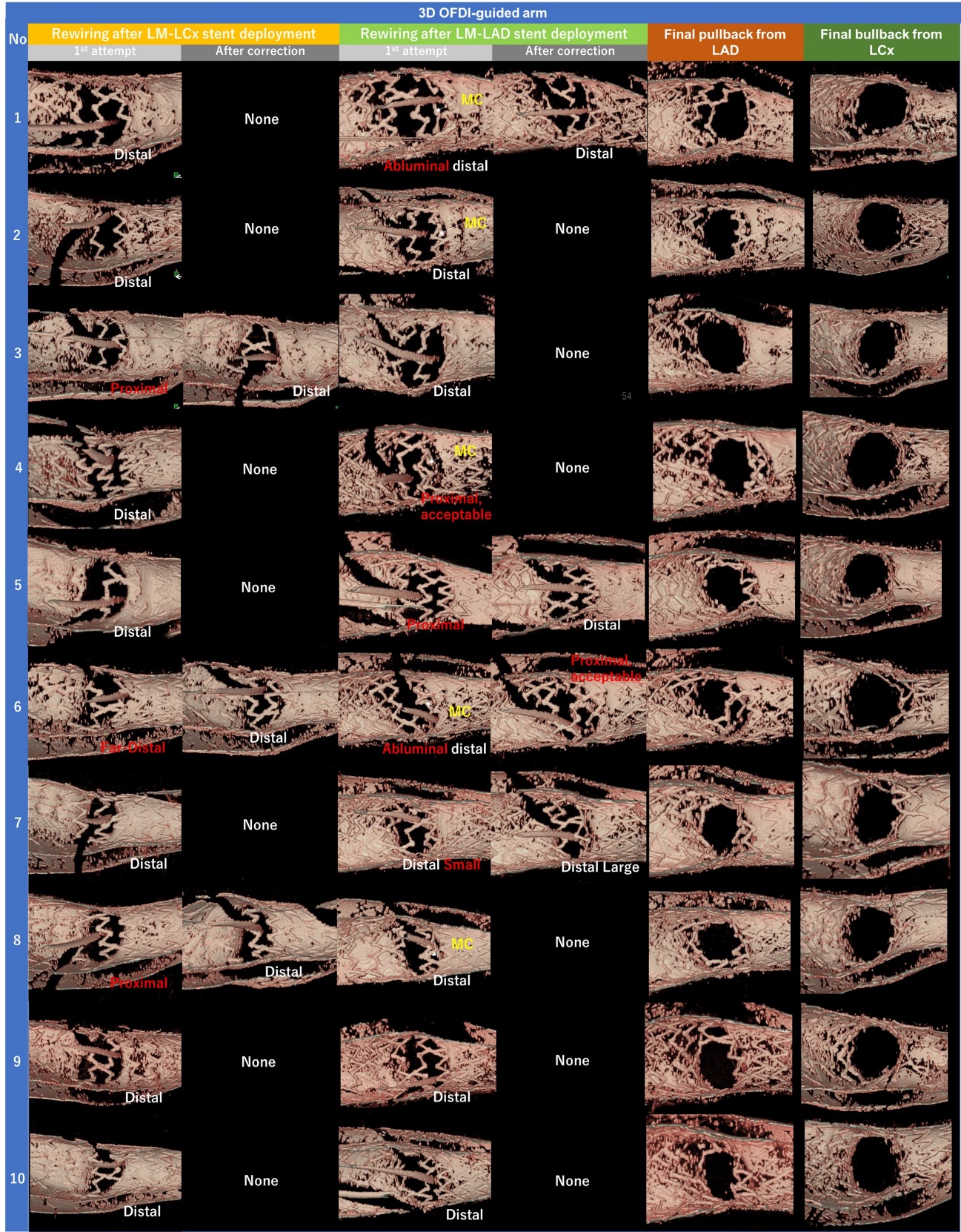
Abbreviations are the same as for Supplementary Figure S1.

**Supplementary Table S1. Vessel diameters and bifurcation angles derived by quantitative coronary angiography**

|  | Actual angle and diameter | Stationary model | Novel LMB  model |
| --- | --- | --- | --- |
| Projection | - | AP | LAO 17°, CAUD 41° |
| Foreshortening ratio | 1.0 | 0.99 | 0.89 |
| LM-LAD angle, ° | 126 | 126.2 | 116.6 |
| LM-LCx angle, ° | 140 | 152.2 | 132.9 |
| LAD-LCx angle, ° | 81 | 88.2 | 118.4 |
| LM diameter, mm | 4.50(5.04x3.64) | 4.81 | 4.79 |
| LAD diameter, mm | 3.20(3.37x2.80) | 3.18 | 2.98 |
| LCx diameter, mm | 2.50(2.85x2.24) | 2.51 | 2.51 |

LM: left main, LAD: left anterior descending coronary artery, LCx: left circumflex coronary artery, LMB: left main bifurcation, AP: anterior posterior, LAO: left anterior oblique, CAUD: caudal.
